# The role of E2A in ATPR‐induced cell differentiation and cycle arrest in acute myeloid leukaemia cells

**DOI:** 10.1111/jcmm.17166

**Published:** 2022-01-09

**Authors:** Meiju Zhang, Long‐fei Wang, Xiaoling Xu, Yan Du, Lanlan Li, Ge Deng, Yubin Feng, Ziyao Ou, Ke Wang, Yayun Xu, Xiaoqing Peng, Feihu Chen

**Affiliations:** ^1^ Inflammation and Immune Mediated Diseases Laboratory of Anhui Province School of Pharmacy Anhui Medical University Hefei Anhui China

**Keywords:** acute myeloid leukaemia, c‐Myc, cycle arrest, differentiation, E2A, P53 pathway

## Abstract

Acute myeloid leukaemia (AML) is a biologically heterogeneous disease with an overall poor prognosis; thus, novel therapeutic approaches are needed. Our previous studies showed that 4‐amino‐2‐trifluoromethyl‐phenyl retinate (ATPR), a new derivative of all‐trans retinoic acid (ATRA), could induce AML cell differentiation and cycle arrest. The current study aimed to determine the potential pharmacological mechanisms of ATPR therapies against AML. Our findings showed that E2A was overexpressed in AML specimens and cell lines, and mediate AML development by inactivating the P53 pathway. The findings indicated that E2A expression and activity decreased with ATPR treatment. Furthermore, we determined that *E2A* inhibition could enhance the effect of ATPR‐induced AML cell differentiation and cycle arrest, whereas E2A overexpression could reverse this effect, suggesting that the E2A gene plays a crucial role in AML. We identified P53 and c‐Myc were downstream pathways and targets for silencing E2A cells using RNA sequencing, which are involved in the progression of AML. Taken together, these results confirmed that ATPR inhibited the expression of E2A/c‐My*c*, which led to the activation of the P53 pathway, and induced cell differentiation and cycle arrest in AML.

## 1 INTRODUCTION

Acute myeloid leukaemia (AML) is a malignant haematopoietic system disease^1^ rooted in aberrant bone marrow cells, and its clinical manifestations include fever, frequent infection,^2^ easy bleeding and bruising.^3^ According to the latest data published by CA: A Cancer Journal for clinicians, the incidence of AML has been increasing worldwide in the past 20 years, whereas the 5‐year survival rate for adults is only 30%.^4^ The mechanisms of AML pathogenesis involve specific chromosome translocations most commonly involving t (15;17) and t (8;21) ect^5^, ^6^ and the pathogenesis pathways are associated with the P53 and AMPK signalling pathways.^7^

**FIGURE 1:**
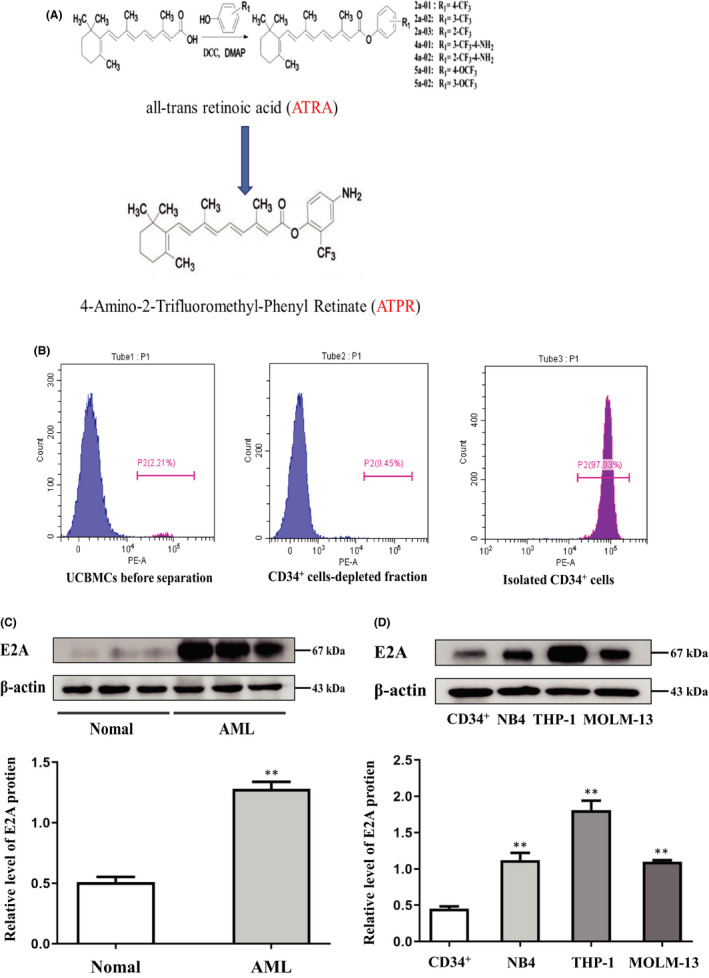
High E2A expression in AML cell lines and patient samples. (A) Structure of new derivative 4‐amino‐2‐trifloromethyl‐phenyl retinate (ATPR) of all‐trans retinoic acid (ATRA) (B) CD34^+^ cells was purified from cord blood and identified by CytoFLEX. (C) Western blotting analysis of E2A expression in AML specimens and normal control. (D) Western blotting analysis of E2A expression in AML cell lines (NB4, THP‐1 and MOML‐13) and CD34^+^ cells. Values were presented as mean ± SD of three independent experiments. *p < .05, **p < .01 versus control group

The most commonly used induction treatment regimen for AML is cytarabine combined with anthracycline agents and stem cell transplantation; however, severe toxic effects, high rates of relapse and resistance are limitations to its application for clinical use.^8^, ^9^ In the late 1980s, all‐trans retinoic acid (ATRA) was used clinically in patients with acute promyelocytic leukaemia (APL), providing the first successful cure case.^10^ However, ATRA has limited efficacy in other AML subtypes, and serious side effects such as retinoic acid syndrome and drug resistance during the treatment process limit its application.^11^ Therefore, AML therapy warrants the development of other highly effective and safe drugs. Our group via structural modification synthesized a new derivative 4‐amino‐2‐trifluoromethyl‐phenyl retinate (ATPR) of ATRA (Figure 1A). Previous studies have confirmed enhanced therapeutic effects on human gastric cancer, hepatocellular carcinoma, gastric carcinoma, breast cancer and leukaemia from ATPR compared with ATRA.^12^ Our group studies demonstrated its anti‐cancer mechanism through the effect on enzyme activity, protein interaction, non‐coding RNA downstream regulation, etc.^11^, ^12^, ^13^ However, many other mechanisms need to be further explored.

As a member of the basic helix–loop–helix transcription factors (BHLH), E2A is located in the cytoplasm and regulates genes related to cell proliferation and differentiation.^14^ Its BHLH domain mediates the dimer and binds to the E‐box within DNA, and the Ad1/Ad2 domains recruit p300 and GCN5 to enhance the activation of target genes.^15^ E2A has been reported to be highly expressed in a variety of tumour cells, such as cervical carcinoma,^16^ nasopharyngeal carcinoma^17^ and is associated with poor prognosis. The E2A gene is a common target of chromosomal translocation in leukaemia and can easily produce fusion proteins, such as E2A‐PBX1^18^ and E2A‐HLF,^19^ but the mechanism of its regulation in leukaemia has not been clarified. Taken together, these results suggest that alteration of the E2A gene may play a key role in the pathogenesis of leukaemia.

To further explore the role of E2A in AML, we performed transcriptome sequencing, and the results showed that silencing E2A could specifically downregulate c‐Myc. c‐Myc regulates cell growth, differentiation and apoptosis, and it is also present in the nucleus of cells and highly expressed in a variety of human tumours.^20^ Therefore, we hypothesized that E2A promotes cell proliferation by upregulating the expression of the c‐Myc gene in AML cells. Studies have suggested that c‐Myc overexpression could reverse the activation of the p53 pathway caused by silencing the SNRPG gene in glioma cells, and promoted the development of glioma.^21^ In a variety of tumours, the p53 pathway participates in the regulation of biological processes, including cell cycle arrest and apoptosis, associated with cancer such as leukaemia and liver cancer.^22^, ^23^ In conclusion, we proposed a hypothesis that ATPR inhibits E2A expression in AML cells by interacting with retinoic acid receptor alpha (RARα), thereby reducing the expression of the downstream target gene c‐Myc, activating the P53 pathway and inducing cell differentiation and cycle arrest.

## 2 MATERIALS AND METHODS

### 2.1 Materials

The design and synthesis of ATPR were performed as shown in Figure 1. ATRA was purchased from Sigma‐Aldrich (USA). ATPR and ATRA were dissolved in absolute ethanol to make a stock of 10^−2^ M solution and placed at −20°C.

### 2.2 Samples

Peripheral blood samples were obtained from 15 newly diagnosed AML patients and 20 healthy individuals at the First Affiliated Hospital of Anhui Medical University. Peripheral blood mononuclear cells (PBMCs) were isolated from each sample using standard Ficoll lymphocyte separation solution, and then human CD34^+^ cells were separated from healthy individuals using the CD34 Magnetic Bead Separation kit (Miltenyi Biotec Inc.) following the manufacturer's instructions. All procedures involving human participants in the study were approved by the Ethics Committee of the First Affiliated Hospital of Anhui Medical University and the Declaration of Helsinki.

### 2.3 Cell culture

We purchased human leukaemia cell lines THP‐1 and NB4 from the Shanghai Genechem Co., Ltd. (Shanghai, China) and MOML‐13 cells were obtained from the University of Maryland School of Medicine; the cells were suspended in RPMI‐1640 medium (HyClone) supplemented with 10% foetal bovine serum (FBS) and cultured at 37.5°C in an atmosphere of 5% CO_2_.

E2A knockdown, overexpression and negative control (NC) lentiviruses were synthesized and purchased from Hanbio (Shanghai, China). Twenty microliters of E2A knockdown or overexpression lentiviruses was used to transfect NB4 and THP‐1 cells at a density of 1 × 10^5^ cells/well in 24‐well plates. Three days after lentivirus infection, stable cloned cells were obtained by screening with puromycin (final concentration: 5 μg/mL Meilunbio, Dalian, China) for 2 consecutive days.^24^

The shRNA target sequence for E2A was CCGGCCCGGATCACTCAAGCAATAACTCGAGTTATTGCTTGAGTGATCCGGGTTTTTG, and the negative control (NC) sequence was AATTGAAAAAATTCTCCGAACGTGTCACGTAATCTCTTGAATTACGTGACACGTTCGGAGAACG.

### 2.4 Transcriptome sequencing

Total RNA was extracted from NB4 cells transfected with sh‐NC, and sh‐E2A lentiviral transfection was performed using TRIzol reagent (Invitrogen Corp). The RNA samples were stored at −80°C until analysis. RNA sequencing was performed by the Wuhan Bioacme Biological Technology Co., Ltd.

### 2.5 Western blot analysis

The cells were separated into groups following the designated treatments, washed twice with prechilled phosphate‐buffered saline (PBS) and lysed in RIPA buffer containing PMSF for 30 min for western blotting (Beyotime, China). After centrifugation at 12,000 × *g* for 30 min at 4°C, the supernatant was collected and the total protein concentration was determined using the BCA protein assay kit. An equal amount of protein (20 µg) was obtained from each sample, separated using 10% dodecyl sulphate and sodium salt/polyacrylamide gel electrophoresis, and then blotted onto PVDF membranes (Millipore, Billerica, MA, USA). They were blocked in 5% skim milk for 2 h, and then incubated with the primary antibody at 4°C for 24 h. Then, incubated with the secondary antibody for 1 h at room temperature, and the protein signals were visualized using an enhanced chemiluminescence kit (ECL‐plus; Thermo Fisher Scientific). Primary antibodies against cyclin A2, P53, P‐P53, CDK4, P‐rb, cyclin D3, c‐Myc, CD11b, CD14 (Abcam) and E2A (Proteintech) were used at a dilution of 1:1000. The primary antibody for β‐actin (Bioss, Beijing, China) was used at a 1:500 dilution.

### 2.6 Quantitative real‐time polymerase chain reaction (qRT‐PCR)

Total RNA was isolated from cultured NB4 and THP‐1 cells that were treated with TRIzol reagent (Invitrogen Corp), and reverse transcribed into cDNA using the First Strand cDNA Synthesis Kit (Thermo Fisher Scientific). The relative gene mRNA expression levels in different samples were evaluated via qRT‐PCR using the SYBR‐Green PCR kit (Takara). β‐actin mRNA was used as an internal control for normalization. The sequences of primers used were as follows: β‐actin, 5‐CGCCGCCAGCTCACCATG‐3′ (forward) and 5‐CACGATGGAGGGGAAGACGG‐3′ (reverse); c‐Myc, 5‐GTCAAGAGGCGAACACACAAC‐3 forward) and 5‐TTGGACGGACAGGATGTATGC‐3 (reverse); E2A, 5‐CCGACTCCTACAGTGGGCTA‐3 (forward) and 5‐CGCTGACGTGTTCTCCTCG‐3′ (reverse).

All primers were synthesized and purchased from Sangon Biotech Co., Ltd., and the results were analysed using the 2–ΔΔCT method.

### 2.7 Differentiation marker analysis

The level of maturity of the cells was evaluated via the expression of the cell surface differentiation‐related antigens CD11b and CD14. The treated cells were incubated with 2 µL (CD11b‐PE/CY5; CD14‐FITC) antibody for 30 min in the dark, using a CytoFLEX (Becton Dickinson, USA), and the data were analysed using CytExpert software.

### 2.8 Cell cycle analysis

The treated cells were harvested, washed with prechilled PBS, fixed in 75% ethanol at −20°C overnight and the cell cycle analysis propidium iodide (PI) kit (BestBio, Nanjing, China) was used to detect the intracellular DNA content. The cells were centrifuged at 1000 × *g* for 5 min and washed twice with PBS. The cell samples were suspended in each tube and treated with 20 µL RNase at 37°C in a water bath for 30 min, followed by incubation with 500 µL of PI staining buffer (Beyotime, China) for 30 min at room temperature protected from light. The cell cycle data were acquired to using CytoFLEX (Becton Dickinson, USA), and the ratio of cells in G0/G1, S and G2/M phases was determined using Modfit software (Verity Software House, USA).

### 2.9 Wright–Giemsa staining

The designated cells were separated into several groups, treated with ATPR (1 × 10^−6^ M) for 3 days and washed twice with prechilled PBS. The cells were fixed on glass slides, dried at room temperature and stained with Wright–Giemsa, and morphological differentiation was observed by fluorescence inversion microscope system (OLYMPUS).

### 2.10 Nitroblue tetrazolium (NBT) reduction assay

For the NBT reduction assay, cells were treated as designated and washed with PBS. A 10‐μL aliquot of the mixed solution, containing 10 mg/mL NBT (Sigma‐Aldrich) and 2 μg/mL PMA (Sigma‐Aldrich), was added to each tube and incubated for 30 min at 37°C.

### 2.11 Double immunofluorescent staining

The cells were seeded in a six‐well plate, washed twice with PBS after harvest and fixed with a fixing solution for 1 h at 4°C. After centrifugation at 1000 × *g* for 10 min, the fixed cells were washed twice with PBS. The cells were then permeabilized with 0.5% Triton X‐100 for 10 min and then blocked with 3% bovine serum albumin for 60 min. Next, the cells were incubated with primary antibodies E2A and c‐Myc (Bioss, Beijing, China) (1:200) overnight at 4°C and with the fluorescent‐labelled secondary antibody at 4°C for 1 h, protected from light, followed by counterstaining with 1 μg/mL 4, 6‐diamidino‐2‐phenylindole for 5 min. The images were observed using a fluorescence inversion microscope system (OLYMPUS).

### 2.12 Tumour xenograft

We purchased 6‐ to 8‐week‐old female/male NSG mice from Nanjing Model Animal Research Institute, and randomly divided them into two groups. A tumour xenograft model was produced, and 5 × 10^6^ NB4 cells transfected with sh‐NC and sh‐E2A were subcutaneously injected into the right flank of NSG mice to investigate the effect of E2A on AML in vivo.^25^ The tumour volumes of NSG mice were measured every day, and they were killed 2 weeks later and the tumours were removed for further evaluation.

### 2.13 Histopathology

To analyse the expression of related proteins in tumour tissues from NSG mice, immunohistochemistry (IHC) staining was performed following standard procedures. Human monoclonal E2A, c‐Myc, cyclin A2, CDK4 and CD11b antibodies (Bioss, China) were used for IHC, diluted 1:300 and then detected and photographed using a fluorescence inversion microscope system (OLYMPUS).

### 2.14 Statistical analysis

All statistical analyses were conducted using SPSS software *V*.*21*.*0*, and the data are shown as the mean ± standard deviation. Student's *t*‐test was used to compare two groups, whereas one‐way analysis of variance was used for comparing multiple groups. Statistical significance was set at *p* < 0.05.

## 3 RESULTS

### 3.1 High E2A expression in AML cell lines and patient samples

To determine the expression of the E2A gene in AML, PMBCs of all participants were collected, and CD34^+^ cells were purified from cord blood and identified using CytoFLEX (Figure 1B). We then monitored changes in the levels of E2A protein in AML. Western blotting results showed that the level of E2A protein was significantly higher in AML specimens than in healthy controls (Figure 1C; S1A). Furthermore, we analysed a number of AML cell lines (NB4, MOLM‐13 and THP‐1), and the results showed that E2A protein levels were higher than those of normal human CD34^+^ cells (Figure 1D). These results suggest that high expression of E2A may be associated with the progression of AML.

### 3.2 E2A expression in AML cells was inhibited by ATPR treatment

**FIGURE 2:**
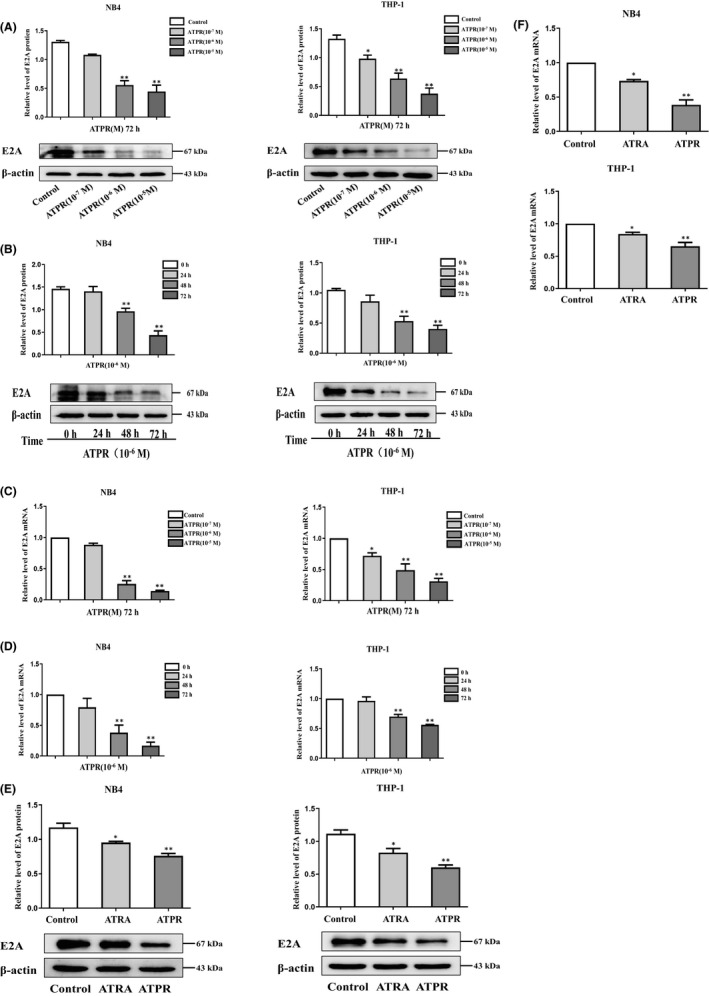
E2A expression in AML cells was inhibited by ATPR treatment. (A) NB4/THP‐1 cells were treated with an ATPR concentration gradient (10^−5^ to 10^−7^ M) for 72 h. (B) NB4/THP‐1 cells were treated with ATPR (10^−6^ M) at different time points (0, 24, 48 and 72 h). Then, the protein expression of E2A was assessed by western blot. β‐Actin was used as the loading control. (C) NB4/THP‐1 cells were treated with ATPR in different the mRNA expression of E2A were analysed by qPCR. (D) NB4/THP‐1 cells were treated with ATPR in different times, and the mRNA expression of E2A was analysed by qPCR. (E) NB4/THP‐1 cells were treated with ATRA and ATPR (10^−6^ M, 72 h), and the protein expression of E2A was assessed by western blotting. (F) NB4/THP‐1 cells were treated with ATRA and ATPR (10^−6^ M, 72 h), the mRNA expression of E2A was analysed by qPCR. All the data are expressed in mean ± SD of three independent experiments. *p < 0.05, **p < 0.01, vs. control group

To further analyse the effect of the E2A gene on ATPR treatment of AML, we first used ATPR at different concentrations (10^−5^–10^−7^ M) at different times (0, 24, 48 and 72 h) in AML cells. We found that the level of E2A protein in AML cells was significantly decreased after 72 h of treatment with 10^−6^ M ATPR (Figure 2A, B). As shown in Figure 2C and D, ATPR significantly reduced E2A mRNA levels in a dose‐ and time‐dependent manner. Furthermore, in AML cells treated with ATRA or ATPR alone, we observed that the expression of E2A in cells treated with ATPR was decreased (Figure 2E, F). Together, these results indicate that ATPR induces the degradation of E2A in AML cells.

### 3.3 ATPR‐induced AML cell differentiation and cycle arrest was enhanced in the absence of E2A

**FIGURE 3:**
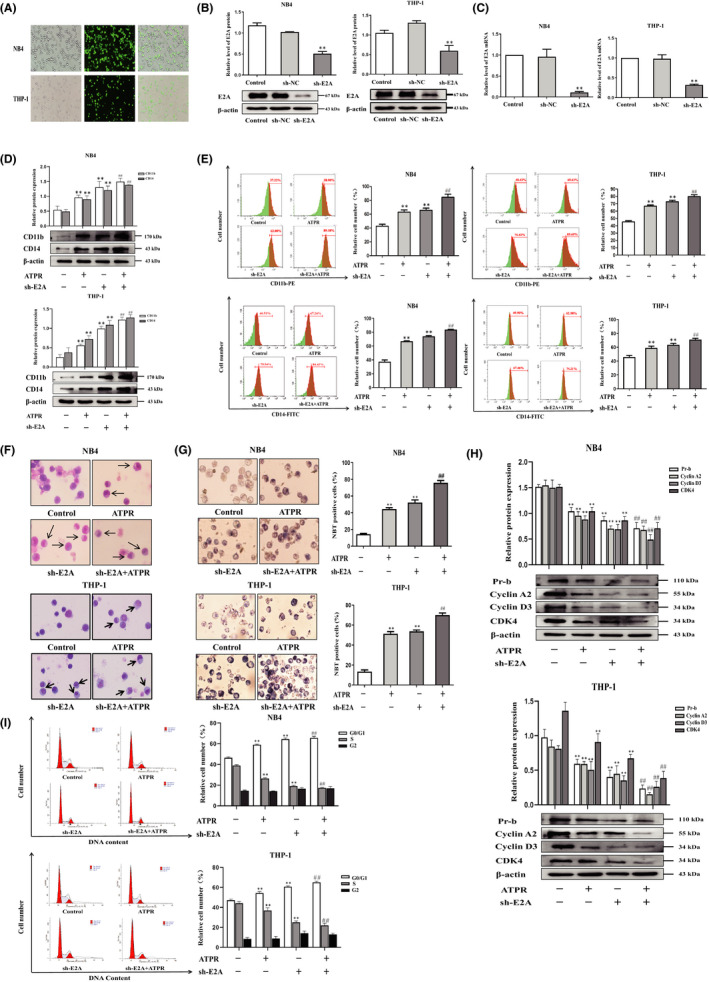
ATPR‐induced AML cell differentiation and cycle arrest were enhanced in the absence of E2A. NB4/THP‐1 cells were transfected with lentivirus containing E2A shRNA to downregulated E2A expression. After cells were exposed to ATPR (10^−6^ M) for another 72 h, a series of independent experiments were conducted as follows. (A) The stable control and sh‐E2A‐transfected NB4/THP‐1 cells were observed by an inversed fluorescent microscope. (B) After treatment with sh‐E2A for 3 days, the protein expression of E2A was assessed by western blotting. (C) After treatment with sh‐E2A for 3 days, the mRNA expression of E2A was assessed by qPCR. (D) After E2A was silenced, cell surface differentiation antigens CD11b and CD14 levels were assessed by western blotting. (E) After E2A was silenced, cell differentiation status was further measured using flow cytometry analysis. (F) After E2A was silenced, cell morphological assays were assessed by Wright–Giemsa staining. Arrows indicate cells with matured morphology, which exhibit kidney‐shaped nucleus and decreased nuclear/cytoplasm ratio. (G) After E2A was silenced, the inversed fluorescent microscope was used to observe the positive cell by NBT reduction experiment. (H) The protein expression of Pr‐b, cyclin D3, cyclin A2 and CDK4 was determined by western blotting analysis. (I) The distribution of cell cycle was analysed by flow cytometer. Data are represented as the mean ± SD. of three independent experiments. *p < 0.05, **p < 0.01 versus negative control group. #*p* < 0.05, ##*p* < 0.01 vs. ATPR group

To evaluate the role of E2A in ATPR‐induced cell differentiation, we used primary AML cells were harvested directly from patients with AML, and analysed by flow cytometry after ATPR treatment, and showed that increased the expression of differentiation markers CD11b/CD14, indicating that ATPR‐induced primary AML cell differentiation (Figure S2A). Next, we transfected sh‐E2A into AML cells to establish AML/sh‐E2A stable clone cells and used sh‐NC transfected into the cells as the control (Figure 3A). Western blotting and qRT‐PCR results showed that, compared with the control group, sh‐E2A cells significantly reduced the levels of E2A protein and mRNA (Figure 3B, C). Western blotting was used to assess the expression levels of cell surface differentiation antigens (CD11b and CD14), and the results revealed that the absence of E2A indeed enhanced the expression levels of CD11b and CD14 proteins induced by ATPR (Figure 3D). Flow cytometry analysis further demonstrated that ATPR‐induced AML cell differentiation was enhanced by the loss of E2A (Figure 3E). Additionally, Wright‐Giemsa staining analysis revealed that the AML cells pretreated with the lentivirus group exhibited a more significant maturity, and the appearance of kidney‐shaped nuclei and decreased nuclear/cytoplasm ratio could be detected compared with ATPR treatment alone (Figure 3F). NBT staining analysis also confirmed these results (Figure 3G). Differentiation was usually accompanied by cycle arrest, so we performed western blotting and demonstrated that the decrease in ATPR‐induced cell cycle‐related proteins (cyclin D3, cyclin A2, Pr‐b and CDK4) was enhanced after pretreatment with sh‐E2A lentivirus (Figure 3H). Similarly, the results of flow cytometry analysis showed that compared with ATPR treatment alone, the combined treatment of sh‐E2A and ATPR revealed a significant increase in the number of arrested cells at the G0/G1 phase and a significant decrease in the S phase (Figure 3I). Taken together, these results indicate that ATPR‐induced AML cell differentiation and cycle arrest were further enhanced in the absence of E2A.

### 3.4 Overexpression of E2A reversed ATPR‐induced differentiation and cycle arrest of AML cells

**FIGURE 4:**
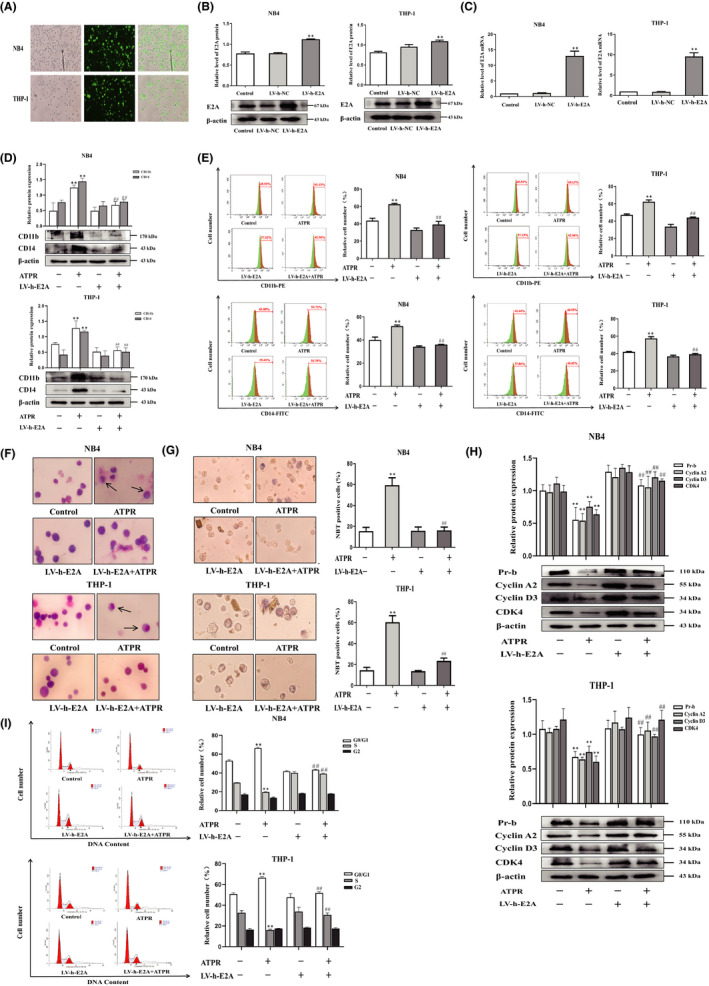
Overexpression of E2A reversed ATPR‐induced differentiation and cycle arrest of AML cells. NB4/THP‐1 cells were transfected with lentivirus containing E2A LV‐h‐RNA to upregulated E2A expression. After cells were exposed to ATPR (10^−6^ M) for another 72 h, a series of independent experiments were conducted as follows. (A) The stable control and LV‐h‐E2A‐transfected NB4/THP‐1 cells were observed by an inversed fluorescent microscope. (B) After treatment with LV‐h‐E2A for 3 days, the protein expression of E2A was assessed by western blotting. (C) After treatment with LV‐h‐E2A for 3 days, the mRNA expression of E2A was assessed by qPCR. (D) After E2A was overexpressed, cell surface differentiation antigens CD11b and CD14 levels were assessed by western blotting. (E) After E2A was overexpressed, cell differentiation status was further measured using flow cytometry analysis. (F) After E2A was overexpressed, cell morphological assays were assessed by Wright–Giemsa staining. Arrows indicate cells with matured morphology, which exhibit kidney‐shaped nucleus and decreased nuclear/cytoplasm ratio. (G) After E2A was overexpressed, and the inversed fluorescent microscope was used to observe the positive cell by NBT reduction experiment. (H) The protein expression of Pr‐b, cyclin D3, cyclin A2 and CDK4 was determined by western blotting analysis. (I) The distribution of cell cycle was analysed by flow cytometer. Data are represented as the mean ± SD. of three independent experiments. *p < 0.05, **p < 0.01 vs. negative control group. #*p* < 0.05, ##*p* < 0.01 versus ATPR group

To investigate the role of E2A in ATPR‐induced differentiation of AML cells, we applied the LV‐h‐E2A lentiviruses to the overexpression of E2A in cells (Figure 4A). Western blotting and qRT‐PCR assays showed that LV‐h‐E2A cells significantly increased the levels of E2A protein and mRNA compared to the control group (Figure 4B, C). Western blotting results revealed that the upregulation of CD11b and CD14 protein expression levels induced by ATPR was reversed by overexpression of E2A (Figure 4D). Flow cytometry analysis further proved that the combined treatment of LV‐h‐E2A and ATPR reversed ATPR‐induced differentiation in AML cells (Figure 4E). Additionally, Wright–Giemsa staining analysis revealed that high expression of E2A prevented ATPR‐induced differentiation of mature granulocytes (Figure 4F). NBT staining results also showed that overexpression of E2A significantly reversed the ATPR‐induced increase in NBT‐positive cells (Figure 4G). These results demonstrate that ATPR‐induced AML cell differentiation is weakened during the overexpression of E2A. In order to reveal the effects of E2A on ATPR‐induced cell cycle arrest, western blotting results demonstrated that ATPR‐induced decreased expression of cyclin D3, cyclin A2, Pr‐b and CDK4 was reversed after pretreatment with E2A overexpression lentivirus (Figure 4H). The results of flow cytometry analysis showed that ATPR‐induced cell cycle arrest was reversed by overexpression of E2A in AML cells (Figure 4F). Taken together, these findings indicate that overexpression of E2A could reverse the cell differentiation and cycle arrest induced by ATPR‐induced AML cells.

### 3.5 Identification of E2A response genes

**FIGURE 5:**
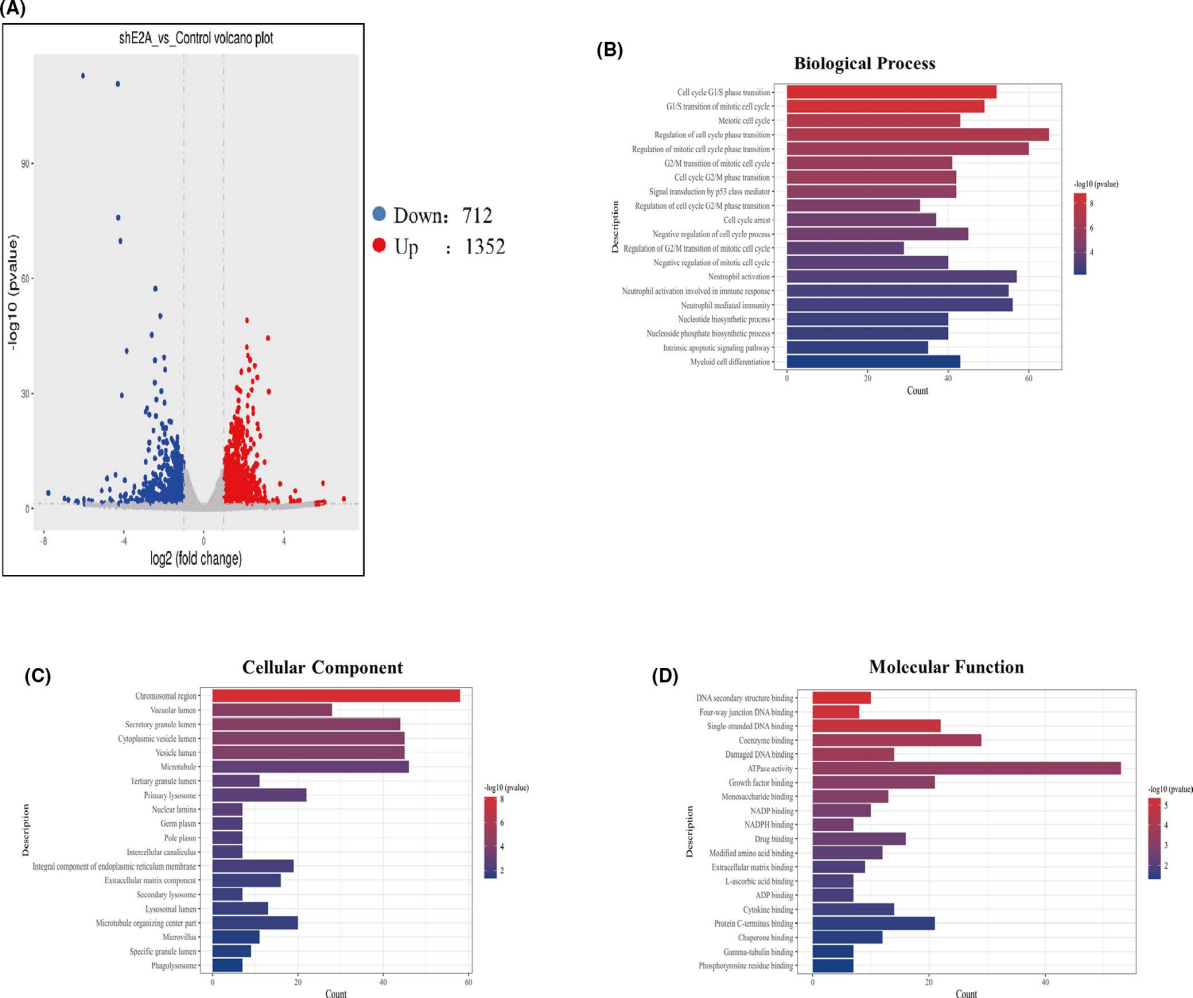
Identification of E2A response genes. (A) Volcano plots of differentially expressed E2A relative to the control group. (B) Top 20 upregulated BP terms during E2A silence in AML cells were ranked by enrichment score. (C) Top 20 upregulated CC terms during E2A silence in AML cells were ranked by enrichment score. (D) Top 20 upregulated MF terms during E2A silence in AML cells were ranked by enrichment score

Based on the above experiments, the effect of sh‐E2A on the differentiation of AML cells was determined. Next, we used the RNA‐seq approach to identify the downstream pathways and targets of sh‐E2A in this process and examined the genome‐wide expression changes during sh‐E2A induction. Microarray analysis was performed to investigate the expression of genes altered by E2A knockout in AML cells, and volcano plots were used to analyse differential expression (Figures 5A and S3). We identified 2641 differentially expressed genes based on fold change ≥2.0, and *p*‐value < 0.05 (*t*‐test). GO analysis revealed that the top 20 most important GO terms were classified and ranked by enrichment scores (*p* < 0.05). The most enriched and significantly upregulated BP terms were associated with the cell cycle, including cell cycle G1/S phase transition, meiotic cell cycle and regulation of cell cycle phase transition (Figure 5B). The most enriched upregulated CC terms were related to chromosomal region, vacuolar lumen, secretory granule lumen and vesicle lumen (Figure 5C). Finally, the most abundant upregulated MF terms were related to DNA secondary structure binding, four‐way junction DNA binding, single‐stranded DNA binding, coenzyme binding and damaged DNA binding (Figure 5D).

### 3.6 E2A drives AML by upregulating c‐Myc and inhibiting the P53 signalling pathway, and ATPR could suppress the E2A/c‐Myc axis

**FIGURE 6:**
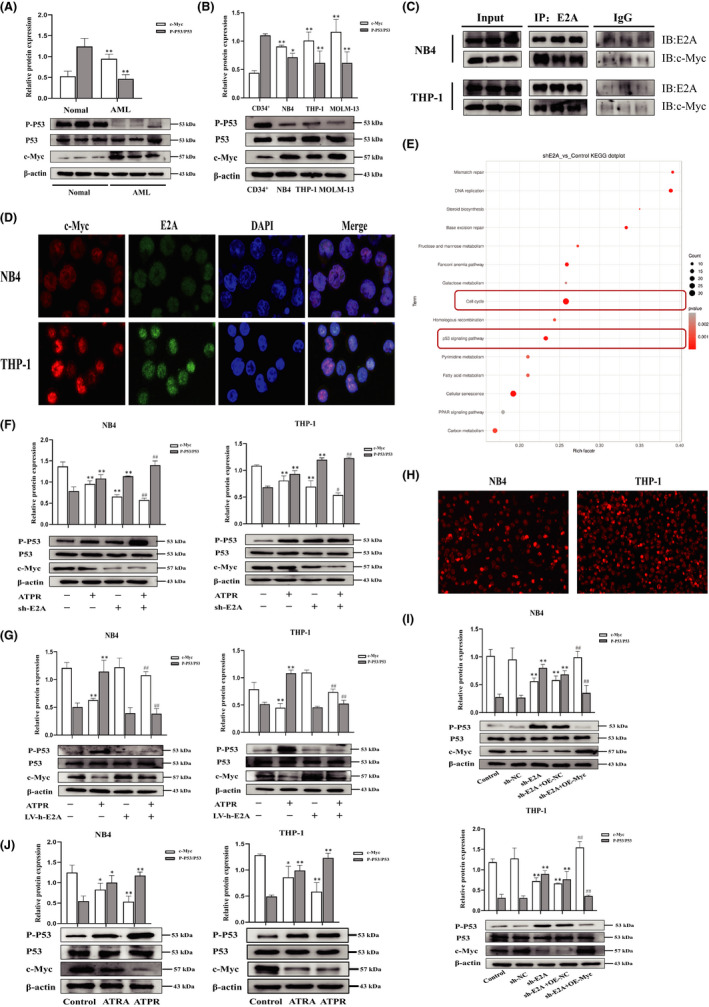
E2A drives AML by upregulating c‐Myc and inhibiting the P53 signalling pathway, and ATPR could suppress the E2A/c‐Myc axis. (A) Western blotting analysis of P53/ P‐P53 and c‐Myc protein levels in AML and normal samples. (B) Western blotting analysis of P53/P‐P53 and c‐Myc expression in AML cell lines (NB4, THP‐1 and MOML‐13) and CD34^+^ cells. (C) The binding of E2A and c‐Myc in immunoprecipitation complex was validated by western blotting. (D) E2A and c‐Myc colocalized in the cytoplasm. (E) KEGG analysis of upregulated pathway genes when E2A was knockout. (F) After E2A was silenced, P53/P‐P53 and c‐Myc expression in AML cells was measured using western blotting analysis. (G) After E2A was overexpression, P53/P‐P53 and c‐Myc expression in AML cells measured using analysis. (H) The OE‐Myc plasmid‐transfected NB4/THP‐1 cells were observed by an inversed fluorescent microscope. (I) The protein levels of p53 were confirmed by western blot in E2A knockdown cells and in plasmid‐infected cells overexpressing c‐Myc. (J) NB4/THP‐1 cells were treated with ATRA and ATPR (10^−6^ M, 72 h), the expression of P53/P‐P53 and c‐Myc was analysed by western blotting. Data are represented as the mean ± SD. of three independent experiments. *p < 0.05, **p < 0.01 vs. negative control group. #*p* < 0.05, ##*p *< 0.01 vs. ATPR group

The oncogenic protein c‐Myc may be a candidate for mediating the effect of E2A on cell differentiation and cycle arrest. P53 is a crucial tumour suppressor protein and deficiency in the majority of AML, which plays a central role in modulating diverse cell cycle and proliferation processes.^26^ Next, we used western blotting to explore the expression of c‐Myc/P53 in the progression of AML compared with control group, and determined that c‐Myc was expressed at higher levels in AML specimens and cell lines, whereas P53 expression was inhibited (Figure 6A, B). c‐Myc was identified as one of the downregulated genes by RNA‐seq after sh‐E2A (Figure 5D), and induced the development of human AML.^27^ Next, by performing Co‐IP analysis, we were confident that E2A could physically bind to c‐Myc in AML cells (Figure 6C). Double immunofluorescence staining revealed that E2A and c‐Myc were localized in the cell nucleus (Figure 6D). The above results demonstrated that E2A interacts with c‐Myc in AML cells. KEGG analysis of upregulated pathway genes, when E2A was knocked out, revealed that the cell cycle, P53 signalling pathway and cellular senescence may play important roles in this process (*p* < 0.05) (Figure 6E). Meanwhile, the data further demonstrated that E2A drives AML by dampening the P53 signalling pathway. Western blotting results showed that sh‐E2A significantly inhibited c‐Myc and activated P53 (Figure 6F), which was reversed by overexpression of E2A (Figure 6G), verifying the regulatory effect of E2A on c‐Myc and P53. Next, we investigated whether the regulation of the P53 signalling pathway by E2A was based on c‐Myc. Western blotting revealed that knockdown of E2A in AML cells resulted in the upregulation of P53, reversed by the c‐Myc overexpression plasmid (Figure 6H, I), further illustrating that E2A regulated the P53 signalling pathway through c‐Myc. Western blotting showed that treatment with ATPR reduced the expression of E2A/c‐Myc, activating the P53 signalling pathway compared to ATRA (Figure 6J). These results indicate that ATPR participates in the treatment of AML by inhibiting E2A/c‐Myc and regulating the P53 signalling pathway.

### 3.7 Loss of E2A inhibits tumorigenesis in vivo

**FIGURE 7:**
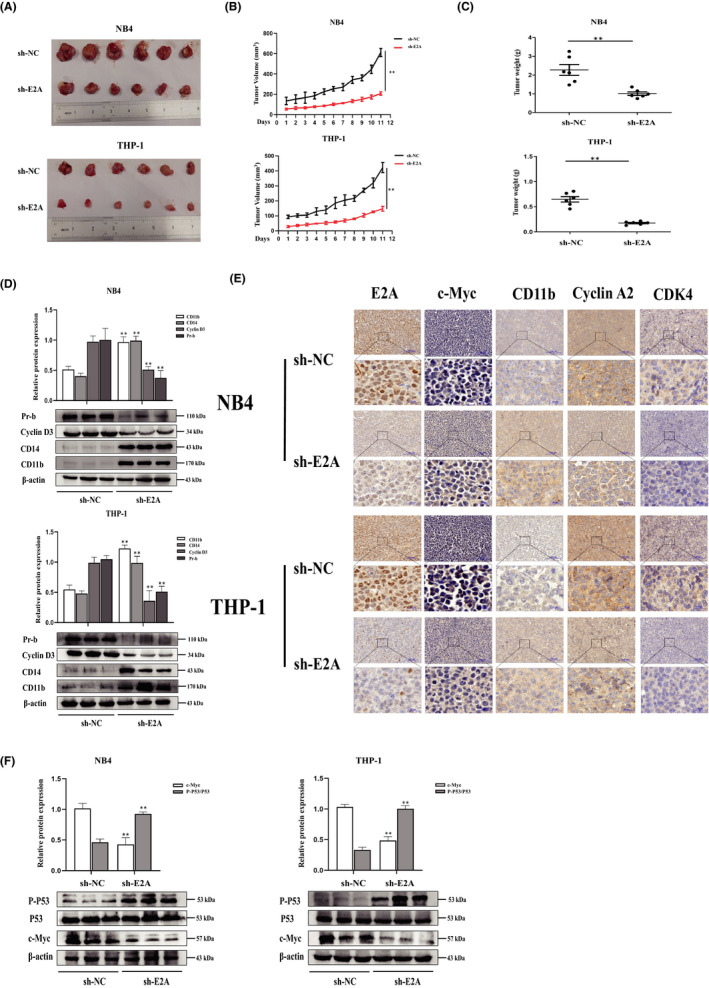
Loss of E2A inhibits tumorigenesis in vivo. 24 NSG mice were randomly divided into four groups (*n* = 6). NB4/THP‐1 cells (5 × 10^6^) transfected with sh‐NC and sh‐E2A were injected subcutaneously in the right shoulder. (A) Tumour images of the xenograft mice were taken at the end of the experiment (*n* = 6 mice per group). (B and C) The tumour volume and weight of xenograft mice were measured during the observation period. (D) Two representative tumour tissues from each group were fixed, and immunohistochemistry staining was performed on E2A, c‐Myc, CD11b, cyclin A2 and CDK4. (E) Western blotting analysis of CD11b, CD14, Pr‐b and cyclin D3 in tumour tissues of sh‐NC and sh‐E2A groups. (F) Western blotting analysis of P‐P53/p53 and c‐Myc in tumour tissues of sh‐NC and sh‐E2A groups. β‐Actin was used as an internal control. Bar graphs (mean ± SD) and representative images are shown. *p < 0.05, **p < 0.01, compared with the NC group

To further substantiate the role of E2A in tumour growth in vivo, shNC/shE2A‐transfected NB4 and THP‐1 cells were subcutaneously injected into NSG mice. The results showed that compared with the control group, the tumours in the sh‐E2A group developed sluggishly (Figure 7A), and the tumour volume and average tumour weight were significantly reduced (Figure 7B and C). Western blotting showed that sh‐E2A significantly increased the levels of CD11b and CD14 proteins and inhibited the expression of cyclin D3 and p‐Rb compared with the control tumours (Figure 7D). IHC staining revealed that the E2A/c‐Myc and cyclin A2/CDK4 genes in the sh‐E2A tumour group were significantly downregulated, whereas the level of differentiation‐related protein CD11b was upregulated (Figure 7E). Meanwhile, western blotting analysis (Figure 7F) also revealed that sh‐E2A increased the expression of P‐P53/P53 and decreased c‐Myc levels, reconfirming that E2A inhibits c‐Myc and regulates the P53 signalling pathway. Collectively, these results indicate that E2A silencing slows tumorigenesis in vivo.

## 4 DISCUSSION

Acute myeloid leukaemia is a heterogeneous disease caused by multiple genetic mutations and cytogenetic abnormalities that involve aetiology, and mechanisms underlying this pathogenesis are extremely complex.^28^ It is characterized by the malignant proliferation and stagnation of differentiation of myeloid progenitor cells (blasts), which result in damage to the immune system and eventually death26. Current treatment methods, such as cytarabine + anthracycline (7 + 3) and stem cell transplantation (Allo‐SCT), have severe side effects and poor tolerance, which do not satisfy the treatment needs of AML27. Therefore, differentiation therapy is a novel treatment for AML. As a classic differentiation‐inducing drug, ATRA has transformed APL from being highly fatal to being highly curable, and the ATRA/arsenic combination programme has cured almost all patients with standard risk APL.^29^, ^30^ Interestingly, the effect of ATRA‐induced differentiation is insufficient for APL eradication, whereas only PML/RARA loss fully extinguish leukaemia‐initiating activity.^31^ Therefore, ATRA/arsenic combination programme exerts an anti‐tumour effect by induced PML/RARA degradation in cells, from which anti‐tumour efficacy could be dissociated from the trancriptomic effect.^32^ This suggests that targeting the degradation of oncoproteins may be a viable therapeutic strategy for some malignancies.

However, ATRA adverse reactions, including drug resistance and retinoic acid syndrome, have promoted the development of safer and more effective therapeutic drugs.^33^ ATPR is a novel derivative of ATRA, which has been proven to have a good anti‐cancer effect on a variety of malignant tumours, and it has the advantages of a longer‐lasting curative effect, higher solubility and lower toxicity than ATRA. Therefore, our study will further explore the mechanism of ATPR‐induced differentiation and cycle arrest in AML cells and provide a theoretical basis for the development of new drugs that induce differentiation. In the current study, the results showed that ATPR inhibited the expression of E2A, thereby reducing the downstream target gene c‐Myc, activating the P53 pathway and inducing AML cell differentiation and cycle arrest.

Studies have reported that the E2A gene regulates cell growth and differentiation and is highly expressed in a variety of tumours.^34^ First, this study confirmed that the expression of E2A in AML patients and cell lines was significantly higher than that in the normal control group. The high expression of E2A may promote the development of leukaemia by upregulating c‐Myc and inhibiting the P53 signalling pathway. In vitro cell experiments revealed that silencing of E2A in AML cells enhanced cell differentiation and cycle arrest induced by ATPR, whereas the overexpression of E2A reversed the therapeutic effect of ATPR. The current experimental results confirmed that ATPR inhibited the expression of E2A/c‐Myc, activated the P53 signalling pathway and induced cell differentiation and cycle arrest.

c‐Myc plays a pivotal role in tumour cell cycle regulation and pathogenesis, and is the central determinant of cell fate, and in diverse types of cancers is dysregulated.^35^ c‐Myc promotes the production of tumour cells by regulating the cell cycle, and the inhibition of c‐Myc is a favourable method for the treatment of cancers, although it promotes intense tumour deterioration.^36^ This study confirmed that, compared with normal controls, the expression level of c‐Myc in AML patients and cell lines was significantly increased. Furthermore, experimental data showed that silencing E2A resulted in a decrease in c‐Myc levels and significantly induced AML cell differentiation and cycle arrest. However, the mechanism by which E2A regulates the expression of c‐Myc requires in‐depth exploration; hence, further research should focus on elucidating the precise mechanism of E2A on c‐Myc.

The P53 signalling pathway plays a significant role in regulating the cell cycle, proliferation and suppressing tumour expression.^37^ Previous studies have indicated that the P53 signalling pathway participates in the regulation of biological processes during normal and leukaemic haematopoiesis.^38^ Transcriptome sequencing data substantiated that the P53 signalling pathway was activated after E2A knockout compared to the control group. Current studies have suggested that silencing E2A could enhance cell cycle arrest and p‐P53 protein levels induced by ATPR; however, the exact mechanism by which E2A regulates the P53 signalling pathway requires further investigation.

**FIGURE 8:**
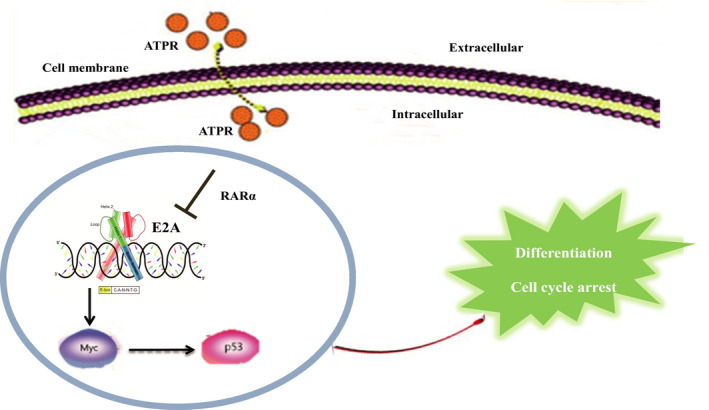
The schematic diagram illustrates ATPR‐induced acute myeloid leukaemia cells differentiation and cycle arrest by blockade of E2A/c‐Myc/P53 pathway. ATPR induces cell differentiation and cycle arrest in vitro and in vivo by inhibiting the expression of E2A/c‐Myc and activating the P53 pathway

Overall, the findings of this study highlight a novel mechanism of ATPR in the treatment of AML. The present study showed that ATPR could downregulate the expression of E2A by binding to RARα, inhibit the downstream target gene c‐Myc and induce cell differentiation and cycle arrest via the P53 signalling pathway (Figure 8). These studies provide evidence that E2A plays a pivotal role in the treatment of AML and provides novel insights for further study of ATPR.

## CONFLICT OF INTEREST

The authors have no conflicts of interest to disclose.

## AUTHOR CONTRIBUTION

**Mei‐ju Zhang:** Conceptualization (equal); Data curation (lead); Formal analysis (lead); Investigation (lead); Methodology (lead); Validation (lead); Writing – original draft (lead); Writing – review & editing (lead). **longfei wang:** Methodology (equal); Validation (equal). **xiaoling xu:** Investigation (equal). **Yan Du:** Writing – original draft (supporting); Writing – review & editing (equal). **Lanlan Li:** Data curation (supporting). **yayun xu:** Data curation (equal). **ziyao ou:** Methodology (supporting). **Yubin Feng:** Software (supporting). **ge deng:** Software (supporting). **Ke Wang:** Methodology (supporting). **Xiaoqing Peng:** Conceptualization (equal). **Feihu Chen:** Conceptualization (lead).

## Supporting information

Figure S1

Figure S2

Figure S3

## Data Availability

The data used to support the findings of this study are included within the article.
