# Supplementary material for: The role of E2A in ATPR‐induced cell differentiation and cycle arrest in acute myeloid leukaemia cells
Source: J Cell Mol Med. 2022 Jan 9;26(4):1128–43. doi: 10.1111/jcmm.17166 (PMC8831953; doi:10.1111/jcmm.17166)
Supplement: Supplementary file 1 — Figure S1 [file JCMM-26-1128-s001.pdf]

Supplementary Figure S1

A

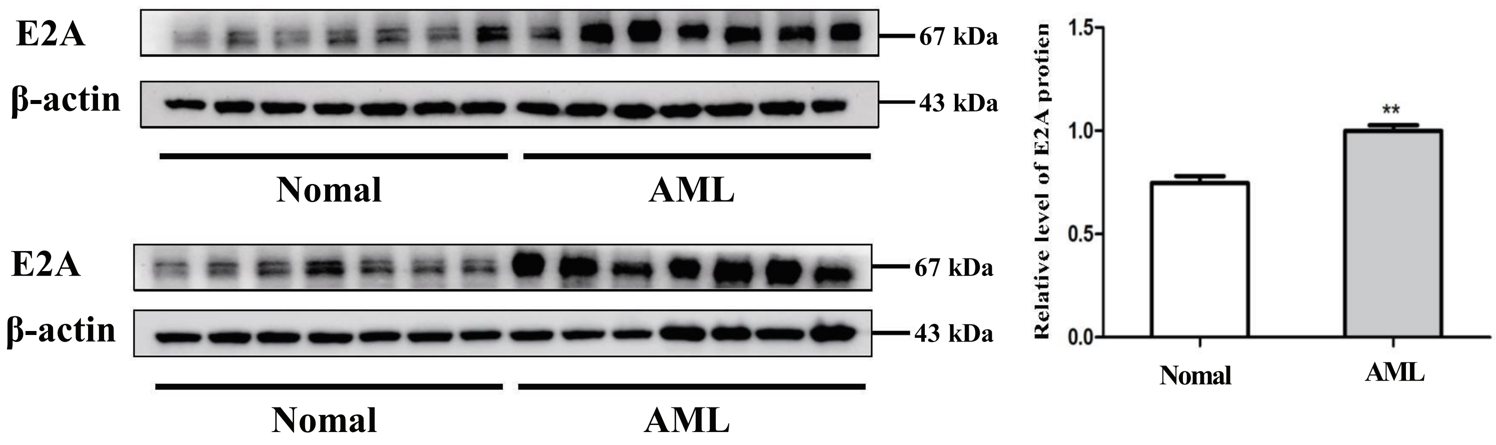

Supplementary Figure S1 High E2A expression in AML cell lines and patient samples. (A) Western blotting analysis of E2A expression in AML specimens and normal control. Values were presented as mean ± SD of three independent experiments. *$p < .05$, **$p < .01$ versus control group.
