# Supplementary figures and images for: The role of E2A in ATPR‐induced cell differentiation and cycle arrest in acute myeloid leukaemia cells
Source: J Cell Mol Med. 2022 Jan 9;26(4):1128–43. doi: 10.1111/jcmm.17166 (PMC8831953; doi:10.1111/jcmm.17166)

Supplementary Figure S2

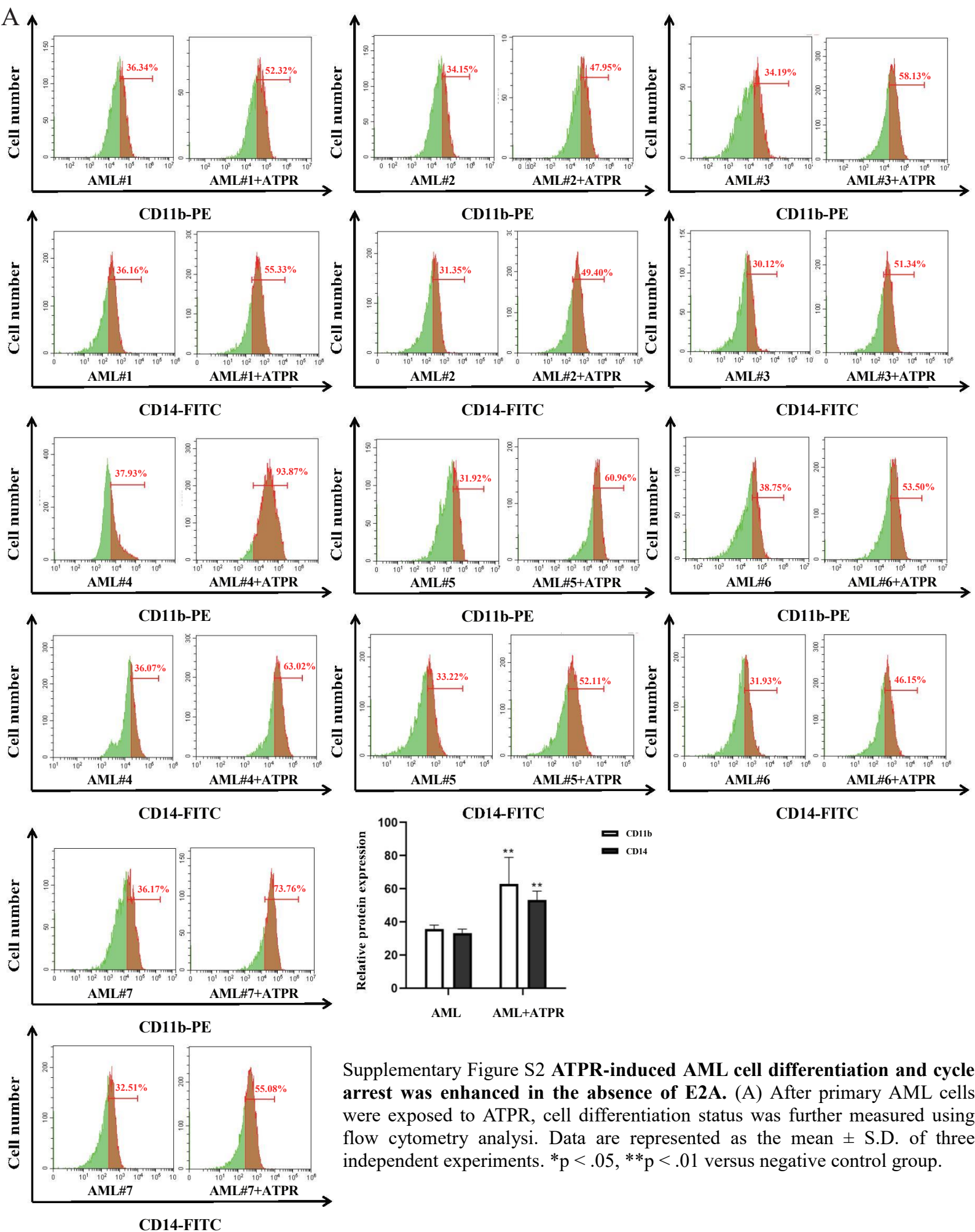

Supplement: Supplementary file 2 — Figure S2 [file JCMM-26-1128-s002.pdf]
