# Supplementary material for: The role of E2A in ATPR‐induced cell differentiation and cycle arrest in acute myeloid leukaemia cells
Source: J Cell Mol Med. 2022 Jan 9;26(4):1128–43. doi: 10.1111/jcmm.17166 (PMC8831953; doi:10.1111/jcmm.17166)
Supplement: Supplementary file 3 — Figure S3 [file JCMM-26-1128-s003.pdf]

# Supplementary S3

A

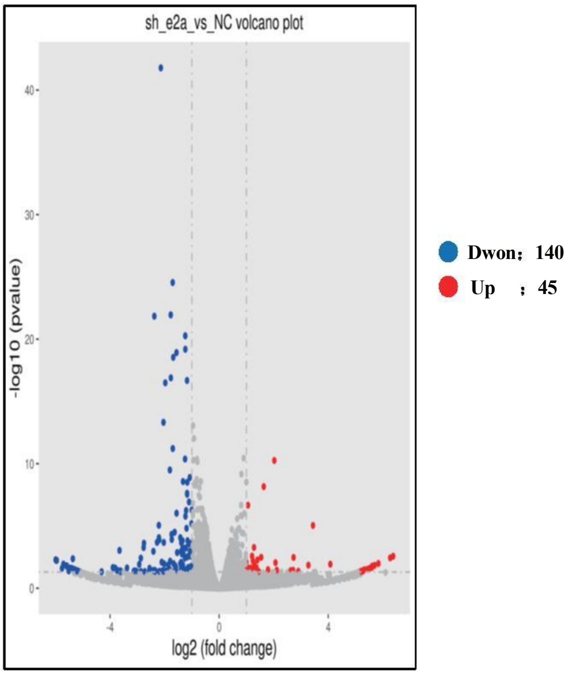


B

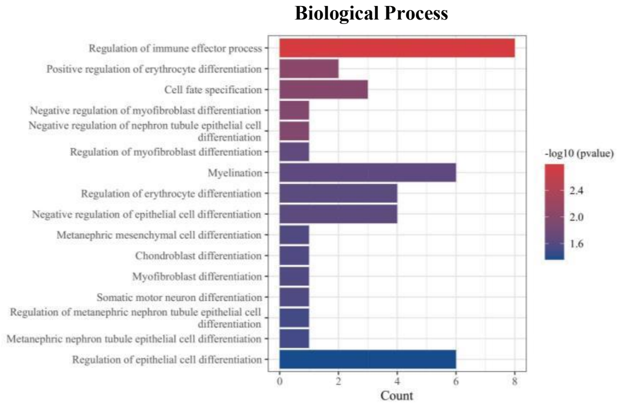


C

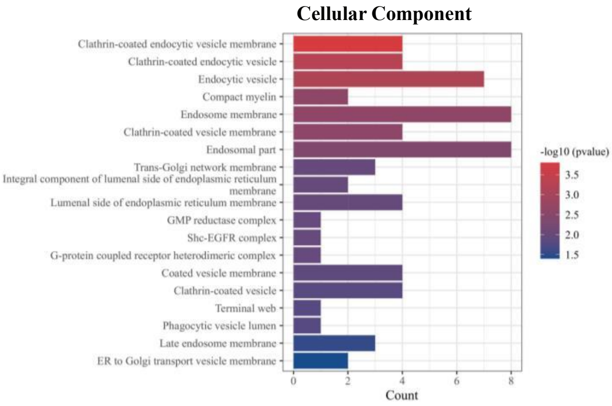


D

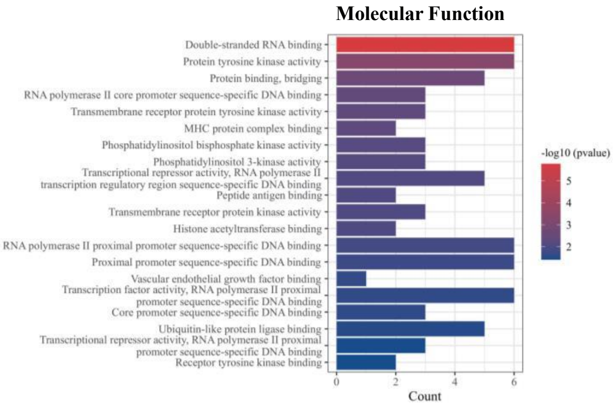


E

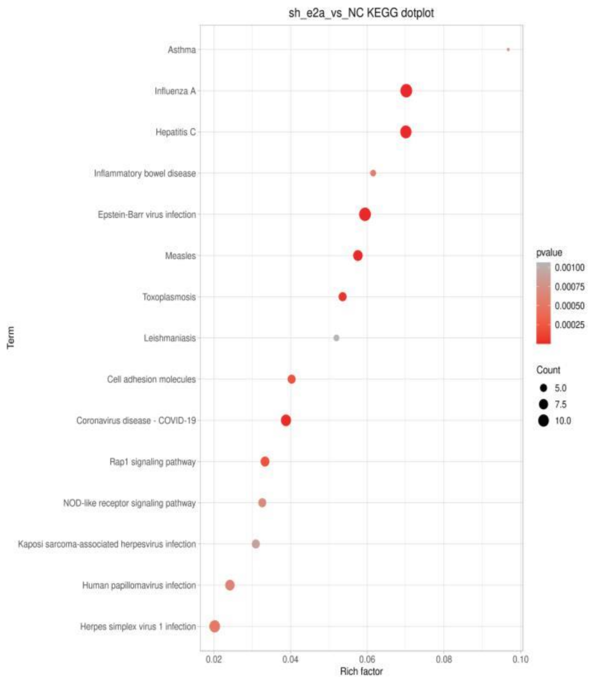


**Supplementary S3 Identification of E2A response genes.** (A) Volcano plots of differentially expressed E2A relative to the control group. (B)Top 20 up-regulated BP terms during E2A silence in AML cells were ranked by enrichment score. (C) Top 20 up-regulated CC terms during E2A silence in AML cells were ranked by enrichment score. (D) Top 20 up-regulated MF terms during E2A silence in AML cells were ranked by enrichment score. (E) KEGG analysis of up-regulated pathway genes when E2A was knockout.
